# Effectiveness of naloxegol in patients with cancer pain suffering from opioid-induced constipation

**DOI:** 10.1007/s00520-021-06299-2

**Published:** 2021-06-13

**Authors:** Antoine Lemaire, Yoann Pointreau, Bérengère Narciso, François-Xavier Piloquet, Viorica Braniste, Jean-Marc Sabaté

**Affiliations:** 1 Oncology and Medical Specialties Department, Valenciennes General Hospital, Valenciennes, France; 2 Oncology and Radiotherapy Department, Inter-regional Institute of Oncology (ILC) – Jean Bernard Center, Le Mans, France; 3 Medical Oncology Department, Bretonneau Regional University Hospital Center, Tours, France; 4 Oncology and Medical Specialties Department, West Oncology Institute – René Gauducheau Center, St. Herblain, France; 5 Medical Department, Kyowa Kirin Pharma, Neuilly-sur-Seine, France; 6 Gastroenterology and Digestive Oncology Department, Avicenne Hospital, AP-HP, Bobigny, France; 7 INSERM U-987, Pathophysiology and Clinical Pharmacology of Pain, Ambroise Paré Hospital, Boulogne-Billancourt, France

**Keywords:** Cancer, Constipation, Naloxegol, Opioids, Pain

## Abstract

**Purpose:**

Naloxegol, an oral once-daily peripherally acting mu-opioid receptor antagonist, is indicated for the treatment of opioid-induced constipation (OIC) with inadequate response to laxative(s), in cancer and non-cancer patients. This study mainly aimed to assess in real-life conditions the efficacy and safety of naloxegol in cancer pain patients and the evolution of their quality of life.

**Methods:**

A non-interventional, 4-week follow-up study was conducted in 24 French oncology and pain centers between 2018 and 2019. Eligible patients were aged ≥ 18 years, treated with opioids for cancer pain, and started naloxegol for OIC with inadequate response to laxatives. The rate of the response to naloxegol (primary criterion) was assessed at W4. The evolution of quality of life was measured using the Patient Assessment of Constipation Quality of Life (PAC-QOL).

**Results:**

A total of 124 patients were included (mean age, 62 ± 12 years; ECOG ≤ 2, 79%; primary cancer, lung 18%, breast 16%, prostate 11%, head and neck 9%, digestive 9%…; metastatic stage, 80%). At inclusion, the median opioid dosage was 60 mg of oral morphine or equivalent. At W4, the response rate was 73.4% (95% CI [63.7–83.2%]), and 62.9% (95% CI [51.5–74.2%]) of patients had a clinically relevant change in quality of life (decrease in PAC-QOL score ≥ 0.5 point). Adverse events related to naloxegol were reported in 8% of patients (7% with gastrointestinal events; one serious diarrhea).

**Conclusion:**

This real-world study shows that naloxegol is effective and well tolerated in cancer pain patients with OIC and that their quality of life improves under treatment.

## Introduction

At some point during their cancer trajectory, up to 90% of the patients will suffer from pain [ \* MERGEFORMAT 1], and most of them will require opioids [ \* MERGEFORMAT 2] which are recommended to control moderate-to-severe cancer pain [ \* MERGEFORMAT 3, \* MERGEFORMAT 4]. However, the therapeutic analgesic effect of opioids can be compromised by non-transient opioid-induced constipation (OIC) [ \* MERGEFORMAT 5] which is their most common side effect, in particular in cancer patients (51% to 87% compared to 41% to 51% of non-cancer patients [ \* MERGEFORMAT 7]). OIC is the result of the binding of opioids to the μ-receptor in the gastrointestinal tract, leading to a decrease in intestinal motility, an increase in fluid and electrolyte absorption in the small intestine and the colon, and an increase in anal sphincter tonus [ \* MERGEFORMAT 8]. Patients with OIC report a significantly worse quality of life compared with non-constipated patients [ \* MERGEFORMAT 5], and constipation may be even more distressing for the patients than the pain itself [ \* MERGEFORMAT 9]. In addition, it is estimated that 30% of patients reduce or stop taking opioids (at least temporary) because of OIC [ \* MERGEFORMAT 10], leading to the risk of compromising analgesia.

Side effects management, such as OIC treatment, belongs to supportive care dynamics that has to be provided to each patient in parallel to specific cancer treatments [ \* MERGEFORMAT 11]. Apart from lifestyle modifications, laxatives are the most common therapeutic strategy for OIC. However, since they do not address directly the underlying cause of OIC, only 50% of the patients are responding to a standard laxative therapy [12]. In this context, a novel class of drugs based on the underlying pathophysiology of OIC has been developed: the peripherally acting mu-opioid receptor antagonists (PAMORAs). The PAMORAs block the peripheral gastrointestinal effects of the opioids without crossing the blood–brain barrier and without affecting their central effects such as analgesia.

Naloxegol is a PEGylated derivate of naloxone created as an oral once-daily PAMORA available in doses of 12.5 and 25 mg. The clinical efficacy and safety of naloxegol were demonstrated in non-cancer pain with OIC in two identical phase III double blind placebo-controlled 12-week studies [ \* MERGEFORMAT 13]. An additional open-label, randomized, parallel-group phase III study conducted in non-cancer patients over a 52-week period showed the good long-term tolerance of naloxegol 25 mg [ \* MERGEFORMAT 14]. One 4-week clinical trial was designed to assess naloxegol in cancer patients, but it had to be early discontinued because of the lack of patient enrollment explained by the too strict selection criteria and the disease severity [ \* MERGEFORMAT 15]. Based on the development program of the drug, naloxegol was approved in 2014 in the European Union for adults with OIC who have an inadequate response to laxative(s) [ \* MERGEFORMAT 16]. However, even if naloxegol is indicated in Europe for the treatment of OIC in non-cancer and cancer pain patients, only few real-life data are available on the drug’s outcomes in cancer patients [ \* MERGEFORMAT 17, \* MERGEFORMAT 18].

The current study was set up in order to assess in real-life conditions the efficacy and safety of naloxegol therapy in cancer pain patients as well as the evolution of their quality of life, constipation, and other symptoms related to OIC.

## Material and methods

### Study design

MovE is a non-interventional prospective French study, with a 4-week follow-up period as recommended by the European Medicines Agency for OIC evaluations in cancer pain patients [ \* MERGEFORMAT 19]. All treatments and assessments were prescribed according to local guidelines and/or routine clinical practice. In accordance with French law regarding non-interventional studies, MovE protocol was approved by an Ethic Committee and was conducted to ensure patient data confidentiality. All patients were informed about the course of the study before enrolment.

### Selection of physicians and patients

Of the 84 specialists regularly prescribing naloxegol for OIC who were invited to participate in the study, 51 (61%) physicians agreed, and 24 (29%) investigators (oncologists, specialists practicing in supportive care centers, pain centers, or palliative care centers) included at least one eligible patient in the study from May 2018 to December 2019. The last patient last visit was performed in January 2020. Eligible patients were aged ≥ 18 years with cancer pain treated with step II or III opioids for their cancer pain, starting naloxegol treatment for OIC with inadequate response to laxative(s), able to complete self-reported questionnaires, and with no objections to participate in the study. Patients participating in an interventional study or with evidence of digestive obstruction were excluded. OIC was defined in accordance with the ROME IV criteria for OIC [ \* MERGEFORMAT 5]. An inadequate response to laxatives was defined by OIC symptoms despite the use of laxatives for at least 4 days prior to inclusion, in accordance with previous international studies [ \* MERGEFORMAT 13].

### Data collection

At the inclusion visit (at naloxegol start), the following data were reported by the physicians: cancer and OIC characteristics, current cancer and pain therapies, and other treatments that could cause constipation, prior and concomitant OIC treatments, patient’s bowel movements over the last 7 days, and OIC symptoms using the Bowel Function Index (BFI) [ \* MERGEFORMAT 20]. The BFI is a clinician-administered, patient-reported, 3-item questionnaire (ease of defecation, feeling of incomplete bowel evacuation, and personal judgment of constipation) to evaluate OIC in cancer and non-cancer chronic pain patients. During the follow-up visit (around 4 weeks after inclusion), additional patient data were collected by physicians: bowel movements over the last 7 days, BFI, changes in opioids and OIC treatments (including naloxegol), the level of satisfaction with naloxegol, and adverse events.

At both visits, patients fulfilled self-reported questionnaires for their OIC assessing symptoms severity (Patient Assessment of Constipation Symptoms, PAC-SYM) [ \* MERGEFORMAT 21] and quality of life (Patient Assessment of Constipation Quality of Life, PAC-QOL) [ \* MERGEFORMAT 22]. The PAC-SYM is a 12-item questionnaire divided into 3 symptom subscales (abdominal, rectal, and stool). The PAC-QOL is a 28-item questionnaire divided into 4 subscales (worries and concerns, physical discomfort, psychosocial discomfort, and satisfaction). As patient perception may be affected by physicians or by the patient wish to give answers they thought in accordance to physician expectations, self-reported questionnaires were fulfilled with no assistance from clinicians and independently returned using provided prepaid envelopes. During the last visit at week 4 (W4), patients also reported their level of satisfaction with naloxegol. Additionally, during the study, the patients completed a diary recording the bowel movements and the naloxegol intake.

### Study size

Based on previous clinical trials having assessed naloxegol in non-cancer patients [ \* MERGEFORMAT 13], the response rate (primary efficacy criterion) was defined as follows for the MovE study: ≥ 3 bowel movements during the 4th week, with or without combined laxatives during follow-up, and an increase of ≥ 1 bowel movement per week between inclusion and W4.

Around 150 assessable patients were expected to describe a response rate of 45% (i.e., a similar proportion to previous findings in non-cancer patients [ \* MERGEFORMAT 13]) with an absolute precision of 8% and an associated confidence interval (CI) of 95%.

### Statistical analysis

Descriptive analysis was performed. All tests were two-sided with α risk at 5%. The 95% CI was provided when relevant. Missing data were not replaced. Statistical analyses were carried out using SAS® software (SAS Institute, NC, USA), version 9.4.

Patient baseline data, effectiveness of naloxegol (response to treatment and evolution of the constipation), and quality of life analysis were analyzed on the population of included patients who met all the selection criteria and received at least one naloxegol tablet (efficacy population).

The analysis of the primary efficacy criterion (response rate in patients with or without concomitant laxatives, based on the data reported by the physicians) was repeated in the subgroups of patients with and without laxatives during study follow-up and using data from the 28-day diaries completed by patients. After univariate analysis performed on baseline patient, cancer, and OIC characteristics, as well as on OIC management, a multivariable, stepwise logistic regression analysis was performed to search for predictive independent factors (p < 0.05) associated with the response to treatment. The evolution of OIC symptoms and quality of life of patients was described in the overall efficacy population and according to the response or not to naloxegol, using the PAC-SYM, BFI, and PAC-QOL scales. The three items of the BFI are scored on numerical 0–100 scales, and the total score in the range of 0–100 corresponds to the mean value; the lower the total score, the lower the symptom burden. A 12-point change in score constitutes a clinically relevant change in constipation [ \* MERGEFORMAT 12]. For PAC-SYM and PAC-QOL, items are scored on 5-point Likert scales, with scores ranging from 0 to 4. A mean total score in the range of 0–4 is generated by dividing the total score by the number of questions completed; the lower the total score, the lower the symptom burden or quality of life impact. The minimal important difference in PAC-SYM and PAC-QOL scores corresponds to a change ≥ 0.5 point [ \* MERGEFORMAT 22, \* MERGEFORMAT 23].

For each patient, the satisfaction with the treatment with naloxegol was assessed by physicians and patients at W4, using a numerical 0–10 scale (from 1, not satisfied at all to 10, very satisfied).

Safety analyses (adverse events, seriousness, and causal relationship with naloxegol) were carried out on the overall population of patients with at least one naloxegol tablet (safety population) and according to the dose prescribed at treatment start (12.5 or 25 mg).

## Results

### Disposition of patients

Of the 133 patients included, 131 patients received at least one dose of naloxegol and were analyzed in the safety population. Of the 124 patients who fulfilled all the selection criteria (efficacy population), 86 patients (69.4%) completed the study at week 4. Early study termination was mainly due to patient death (n = 11), patient decision (n = 7), lost to follow-up (n = 11), or other reasons [n = 9; side effects (3), lack of efficacy (2), and disease progression (2)]. Among the 86 patients who completed the study, 79 were evaluable for the primary criterion (response to naloxegol based on data reported by the physicians).

Regarding patient self-reported questionnaires on constipation symptoms (PAC-SYM) and quality of life (PAC-QOL), 110 were analyzed at inclusion and 78 at W4. In addition, 69 patient diaries on naloxegol intake and bowel movements were analyzed. The response rate to naloxegol could be calculated in 62 of these 69 patients.

### Patients’ baseline characteristics

Baseline characteristics of the patients of the efficacy population are presented in Table 1. Patients frequently suffered from lung, breast, or prostate cancer, with an advanced stage of the disease in the most cases, and two third of them were still treated by either chemotherapy or radiation therapy at inclusion. The median duration of opioid use at baseline was 9.0 weeks (interquartile range (IQR), 2.4–29.1).

**Table 1:** Patient and disease characteristics at inclusion: efficacy population (N = 124)

Baseline parameters	Number of analyzed patients	Efficacy Population N = 124
Demographics		
Age (years), mean ± SD	124	62.1 ± 12.1
Age < 70 years, n (%)	124	95 (76.6%)
Male sex, n (%)	124	117 (63.2)
Body mass index (kg/m^2^), mean ± SD	114	23.8 (4.7)
ECOG index, n (%)	121	
≤ 2		95 (78.5)
> 2		26 (21.5)
Concomitant diseases, n (%)	124	34 (27.4)
Chronic kidney disease		8 (6.5)
Diabetes		6 (4.8)
Hypertension		5 (4.0)
Main cancer locations, n (%)	124	
Lung		22 (17.7)
Breast		20 (16.1)
Prostate		13 (10.5)
Ear, nose, and throat		11 (8.9)
Digestive tract		11 (8.9)
Kidney		8 (6.5)
Bladder		7 (5.6)
Metastases at inclusion, n (%)	100 solid tumors	80 (80.0)
Bones		56 (56.0)
Liver		26 (26.0)
Lung		25 (25.0)
Nodes		15 (15.0)
Current treatment of cancer, n (%)	124	103 (83.1)
Chemotherapy		67 (54.0)
Radiation therapy		19 (15.3)
Immunotherapy		16 (12.9)
Hormonal therapy		11 (8.9)
Targeted therapy		11 (8.9)
Total score of the PAC-SYM at inclusion, mean ± SD	107	2.2 ± 1.2
Total score of the BFI at inclusion, mean ± SD	118	71.2 ± 19.6
Total score of the PAC-QOL at inclusion, mean ± SD	104	2.1 ± 0.6

*BFI*, bowel functional index; *ECOG*, Eastern Cooperative Oncology Group; *PAC-QOL*, Patient Assessment of Constipation Quality of Life; *PAC-SYM*, Patient Assessment of Constipation Symptoms; *SD*, standard deviation

Ongoing opioids were oxycodone (54.8% of patients), morphine (33.9%), fentanyl (29.0%), tramadol (7.3%), opium (2.4%), codeine (1.6%), and methadone (0.8%), with a median dosage of 60 mg (IQR, 22.5–105.0) of oral morphine equivalent at inclusion.

Other treatments that could lead to constipation were also taken at inclusion by 83.5% of patients (grade 1 analgesic, 74.4%; anxiolytic, 33.1%; antidepressant, 27.3%; steroid, 22.3%; anticonvulsant, 18.2%; antispasmodic, 9.1%; anticholinergic, 5.8%).

Prior to inclusion, the median duration of treatment with laxatives was 32.5 days (IQR, 11.0–112.0). At inclusion, patients received osmotically acting laxatives (93.5%), enemas (9.7%), and/or bulk forming laxatives (5.6%). Over the last 7 days prior to inclusion, patients had a mean number of 2.1 ± 1.9 bowel movements.

At inclusion, the median duration of OIC was 4.9 weeks (IQR, 1.6–10.9). The naloxegol starting dose was 25 mg/day in 78.2% of patients, and this dose was used in 82.0% of patients (n = 91) at W4. Over the study period, 76.2% of the patients (n = 64) received at least one concomitant laxative (osmotic-type laxatives in 67.9% of the cases).

In the overall efficacy population, no differences were observed at inclusion between evaluable (n = 79) and not evaluable (n = 45) patients according to the BFI score (p = 0.922) and the PAC-SYM score (p = 0.560).

### Effectiveness

**Fig. 1:**
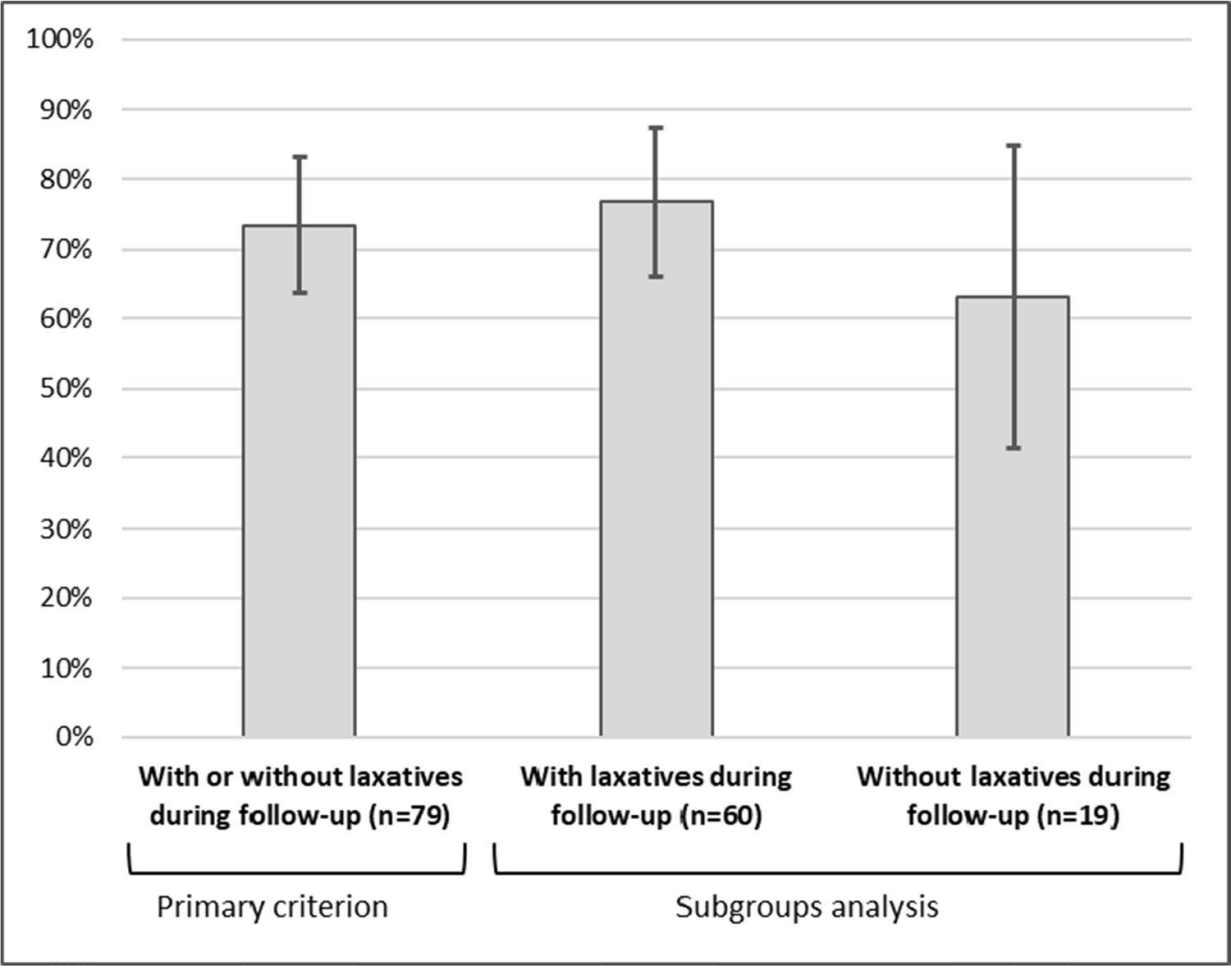
Response to naloxegol at week 4. The response to naloxegol was defined as follows:  ≥ 3 bowel movements during the 4th week after inclusion and an increase from baseline of  ≥ 1 bowel movement per week between inclusion and the 4th week. The proportions of responder patients at the 4th week are graphically presented with their associated confidence intervals

The response to treatment at W4 (primary criterion) was reached by most of the 79 evaluable patients in the efficacy population (73.4%, 95% CI [63.7–83.2%]), irrespective of the use of laxatives during patient follow-up (76.7% [66.0–87.4%] and 63.2% [41.5–84.8%], respectively, with and without laxatives) (Fig. 1). For some of the patients who used laxatives during the 4-week follow-up, another laxative class was added since inclusion (6/64, 9.4%).

Univariate analysis (Table 2) highlighted that the response to naloxegol was associated (p ≤ 0.2) with the following baseline parameters: metastatic cancer, bone metastasis, duration of opioid treatment prior to inclusion, duration of OIC prior to inclusion, number of weekly stools, abdominal symptoms using the PAC-SYM subscale, concomitant analgesics, anxiolytics, antispasmodics, and starting dose of naloxegol. Based on these ten parameters, multivariate analysis (Table 2) showed two baseline parameters were independent predictive factors of treatment response (p < 0.05): duration of OIC less than the median value of 9 weeks (p = 0.006) and bone metastasis (p = 0.047).

**Table 2:** Factors associated with naloxegol response

Baseline parameters	Univariate^a^ OR [95% CI] *p value*	Multivariate^a^ OR [95% CI] *p value*
Metastatic stage of the cancer	2.31 [0.67–8.01]	
	*0.1861*	
Bone metastasis	2.99 [0.97–9.23]	3.62 [1.02–12.82]
	*0.0574*	*0.0473*
Duration of opioids < 9 weeks (median)	5.14 [1.63–16.18]	5.16 [1.59–16.77]
	*0.0051*	*0.0063*
Duration of OIC < 1.12 month (median)	3.14 [1.02–9.66]	
	*0.0455*	
Mean number of tools over the last 7 days	0.71 [0.50–1.01]	
	*0.0584*	
Abdominal symptoms (mean PAC-SYM subscore)	0.59 [0.33–1.07]	
	*0.0808*	
Concomitant analgesics	3.39 [1.16–9.90]	
	*0.0259*	
Concomitant anxiolytics	3.78 [0.99–14.44]	
	*0.0520*	
Concomitant antispasmodics	0.22 [0.03–1.44]	
	*0.1143*	
Start dose of naloxegol at 12.5 mg	0.38 [0.13–1.14]	
	*0.0845*	

*CI*, confidence interval; *OR*, odds ratio; *PAC-SYM*, Patient Assessment of Constipation Symptoms

^**a**^*The factors significant at the 20% level are presented for univariate analysis and entered the final model; factors significant at the 5% level are presented for multivariate analysis*

On the basis of the data from the 62 patients who completed assessable diaries during the 4-week study period, the response rate (with or without laxatives during follow-up) was close to the estimate based on data reported by physicians: 69.4%, 95% CI [57.9–80.8%].

**Fig. 2:**
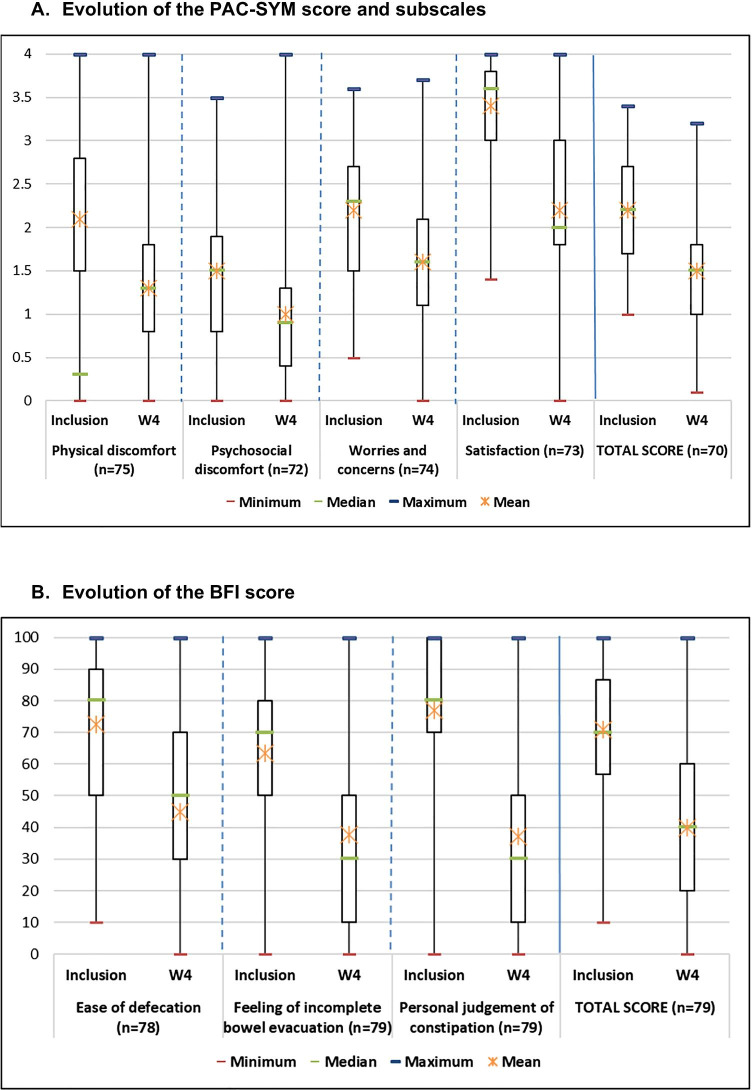
Evolution of the constipation symptoms of patients. **A** Evolution of the PAC-SYM score and subscales. **B** Evolution of the BFI score. BFI, bowel function index; PAC-SYM, patient assessment of constipation symptoms; W, week

Based on diaries completed by patients between inclusion and W4, the mean number of their weekly bowel movements increased from 2.1 ± 1.9 at inclusion to 3.9 ± 2.1 at week 4. A total of 68.7% of patients had a first stool the same day or the day after the first intake of naloxegol. Regarding the other symptoms of constipation, the mean total scores of the 0–4 scale of the PAC-SYM and the 0–100 scale of the BFI decreased (i.e., improved) under treatment, from 2.1 ± 0.7 to 1.3 ± 0.8 (Fig. 2A) and from 70.9 ± 19.2 to 40.0 ± 26.0 (Fig. 2B), respectively, in patients assessable at both visits (inclusion and W4). The strongest improvements were observed for the subscale “stool symptoms of the PAC-SYM” (from 2.6 ± 0.8 to 1.5 ± 0.9) and the subscale “personal judgment of constipation” of the BFI (from 77.0 ± 23.2 to 37.2 ± 28.8). The majority of patients had a clinically relevant change in constipation during follow-up: 70.7% (95% CI [60.4–81.0%]) based on the PAC-SYM and 73.4% (95% CI 63.7–83.2%]) based on the BFI.

**Fig. 3:**
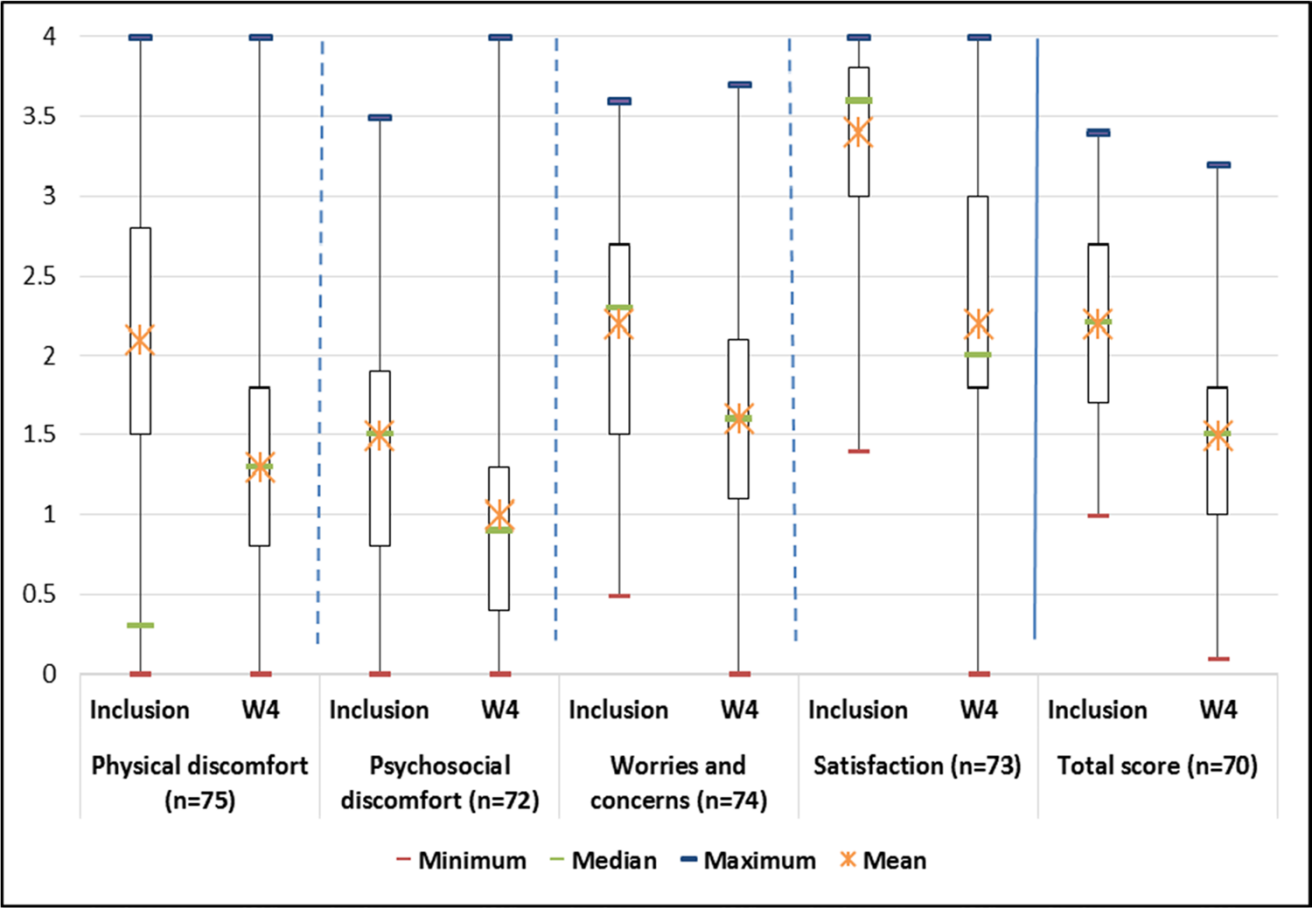
Evolution of the quality of life of patients. w, week

The quality of life of patients improved at W4, with a decrease in PAC-QOL score from 2.2 ± 0.6 to 1.5 ± 0.7 (Fig. 3). Overall, 62.9% of patients (95% CI [51.5–74.2%]) had a clinically relevant change in quality of life.

### Satisfaction with naloxegol treatment

For the analyzed patients, the majority of the physicians and patients themselves were satisfied with the naloxegol treatment at W4: a rating ≥ 5 was observed in 81.4% and 72.4% of the cases, respectively, using a 1–10 numerical scale.

### Safety

At least one adverse event (AE) was reported in 43 patients (32.8%) during the study (median follow-up of patients, 4.3 months; range, 0.3–14.9), including 21 patients (16.0%) with at least one serious AE (SAE). Among the 15 AEs related to naloxegol according to investigators (11 patients, 8.4%), the most common events were gastrointestinal disorders (12 events reported in 9 patients, 6.9%) (Table 3). Among these 12 gastrointestinal AEs, 6 diarrheas were reported in 5 patients (3.8%), all of them having started naloxegol at the dose of 25 mg. One non-serious withdrawal syndrome was reported. Only one related SAE was reported (diarrhea, 0.8% of patients).

**Table 3:** Adverse events related to naloxegol: safety population (N = 131)

		Starting dose of naloxegol		Total N = 131
n (%)		25 mg **N** = 102	12.5 mg **N** = 29	
At least one related adverse event		9 (8.8)	2 (6.9)	11 (8.4)
Gastrointestinal disorders	All	7 (6.9)	2 (6.9)	9 (6.9)
	Diarrhea	5 (4.9)	-	5 (3.8)
	Abdominal pain	1 (1.0)	1 (3.4)	2 (1.5)
	Constipation	-	1 (3.4)	1 (0.8)
	Nausea	1 (1.0)	-	1 (0.8)
	Vomiting	1 (1.0)	-	1 (0.8)
	Eructation	-	1 (3.4)	1 (0.8)
General disorders and administration	All	2 (2.0)	-	2 (1.5)
	Pain	1 (1.0)	-	1 (0.8)
	Withdrawal syndrome	1 (1.0)	-	1 (0.8)
Metabolism and nutrition disorders	All	1 (1.0)	-	1 (0.8)
	Decreased appetite	1 (1.0)	-	1 (0.8)

Thirty AEs experienced by 22 patients (16.8%) led to naloxegol discontinuation during the study, mainly as cancer progression (8 events, 6.1% of patients) and diarrhea (4 events, 3.1% patients, all of them in the 25-mg group).

Thirteen deaths were associated with AEs, without causal relationship with naloxegol as assessed by physicians.

## Discussion

This real-world study evaluated the effectiveness and safety profile of naloxegol in cancer pain patients suffering from OIC with inadequate response to standard laxatives. It showed an improvement in constipation associated with a better quality of life, with an acceptable tolerance in this context.

We observed a 73% overall response rate to naloxegol at week 4. This proportion of responders was higher than in previous clinical trials conducted in non-cancer patients, with no concomitant laxative use permitted (48.7% and 46.8% at week 12 in the KODIAC-04 and KODIAC-05 studies [ \* MERGEFORMAT 13]). Even if it is recommended in Europe that all currently used maintenance laxative therapy should be halted when naloxegol therapy is initiated [ \* MERGEFORMAT 16], in our real-world study, the majority of the patients received concomitant laxatives during follow-up. This medical practice was consistent with a prospective observational Spanish study evaluating naloxegol in cancer patients who received concomitant laxatives in 63% of the cases [ \* MERGEFORMAT 18]. This therapeutic choice of physicians involved in care of cancer patients may be explained by the multifactorial cause of the constipation that may require treatments with synergetic mechanisms of action. Recent real-world studies conducted in cancer patients with OIC also showed similar high response rates under naloxegol treatment. However, in the study conducted by Cobo et al. in 126 patients over 12 weeks, the authors found no differences depending on whether or not a laxative was taken in combination with naloxegol (88% response rate in both groups) [ \* MERGEFORMAT 18]. On the other hand, preliminary results of a European observational 4-week study conducted in cancer patients with no combined laxatives for the 24 h prior to naloxegol intake showed a proportion of responders of 74% [ \* MERGEFORMAT 17]. Finally, the high overall response rate to naloxegol is constant with available literature data in cancer patients with CIO. The differences in proportion could be explained by potential concomitant laxatives but also by other treatments taken by such cancer patients in a real-life setting (in particular in metastatic patients). In our study, most patients in both groups (with or without combined laxatives) also received treatments that could lead to constipation.

Our high overall response rate based on the evolution of the number or bowel movements was confirmed by the improvement of the constipation symptoms under naloxegol, as more than 70% of the patients had a clinically relevant change in constipation after 4 weeks. In addition, the quality of life of patients also improved with a clinically relevant change in 63% of them. All these positive findings could explain the high degree of satisfaction of physicians and patients, which is consistent with the overall effectiveness of naloxegol we observed and suggest the importance cancer patients attach to the improvement of their constipation and their quality of life.

In our study, a treatment with opioids longer than 9 weeks (median value) was significantly associated with a lower response rate. This duration shorter than in clinical trials (mean > 40 months) could explain the lower response rates observed in interventional studies and could suggest that OIC is more difficult to manage in patients with long opioid treatment. In addition, bone metastasis at inclusion was also shown as an independent predictive factor of treatment response. As no baseline differences were observed between patients with and without bone metastasis, this result is difficult to interpret.

The most commonly reported AEs related to naloxegol were gastrointestinal (mainly as diarrhea), and only one serious diarrhea was reported. Diarrhea appeared to be dose-ordered in frequency, occurring only in the 25-mg group. Similar findings were shown in previous clinical trials conducted in non-cancer patients [ \* MERGEFORMAT 13]. The safety profile observed in our study is then consistent with the safety knowledge of the drug.

The limitations of our study are inherent to its non-interventional design (no control group, studied parameters only analyzed when available in the patient medical files). However, a previous clinical trial assessing naloxegol in cancer patients failed [ \* MERGEFORMAT 15], which has shown the difficulties to conduct an interventional study for supportive care in such patients. Patients enrolled in our real-world study suffered from various primary cancers, often at an advanced stage as previously observed [ \* MERGEFORMAT 17, \* MERGEFORMAT 18], suggesting that our studied population is representative of treated cancer pain patients at naloxegol start.

## Conclusion

Finally, this real-world study provides new information on the efficacy and safety of naloxegol use in cancer patients with OIC and shows in this population a high response rate and improvement in both constipation symptoms and quality of life, with a good tolerability. Future research would be useful, in particular to further knowledge of the characteristics of best treatment responders.

The management of multimorphic cancer pain is one of the fundamental pillars of *supportive care in cancer* and is thus part of a complementary approach to the care specific to cancer [24, 25]. *Supportive care in cancer* is “the prevention and management of the adverse effects of cancer and its treatment. This includes management of both physical and psychological symptoms and adverse events across the continuum of the cancer experience from diagnosis, through anti-cancer treatment, to post-treatment care” [10]. For our part, we retain the fact that the management of cancer pain is an integral part of supportive care as soon as the cancer diagnosis is made. As opioids are an important part of multimorphic cancer pain management with other treatments and interventional or complementary approaches [3, 4, 25], OIC must be taken into account as one of the factors that can decrease quality of life or unbalance our analgesic strategy. Since targeted therapies like naloxegol have proven to be safe and efficient on OIC in cancer patients, we can now benefit from new tools to help us reach the best symptoms management, for the right patient, at the right time.

## Data Availability

The data that support the findings of this study are available from the corresponding author, upon reasonable request.

## Code availability

N/A.
