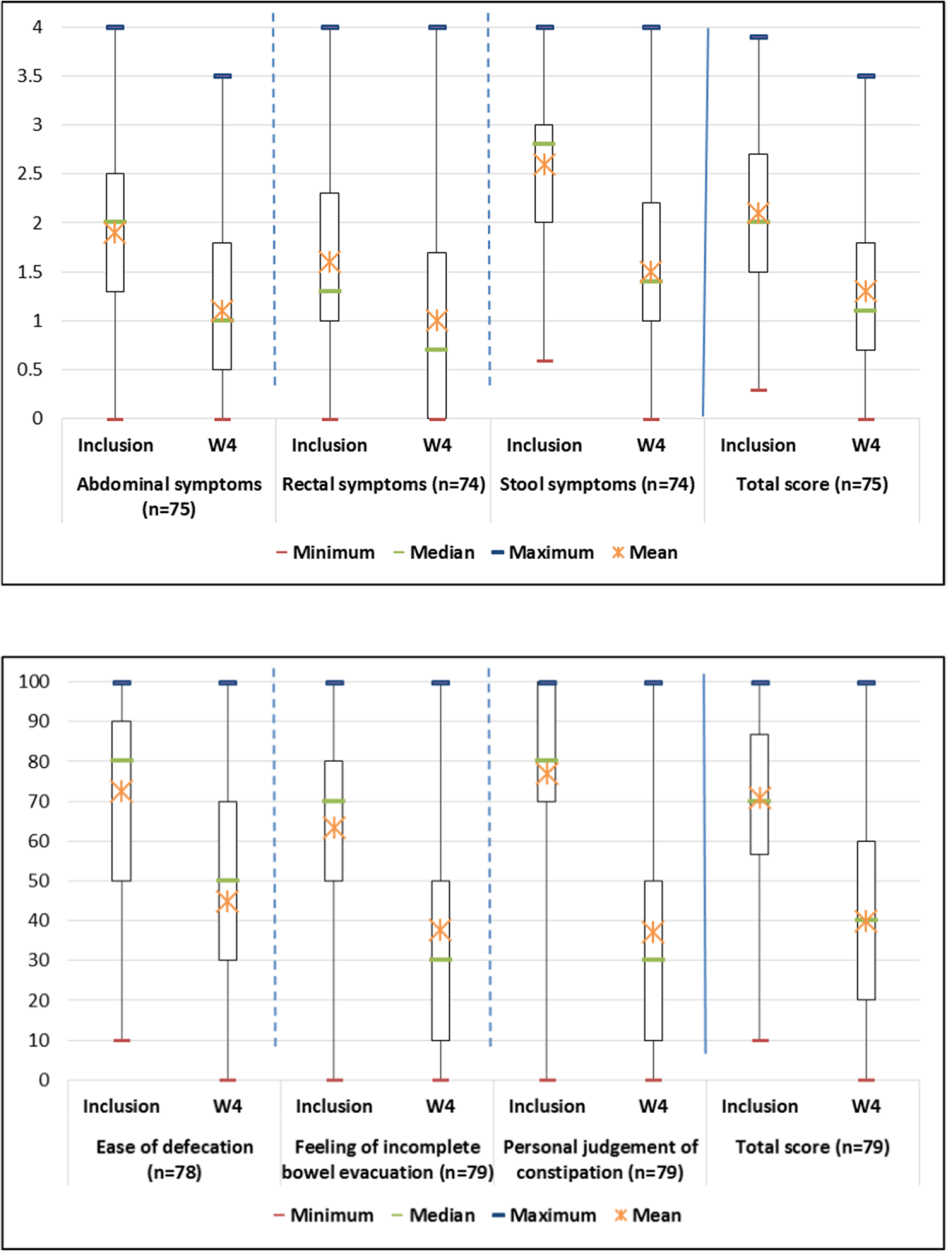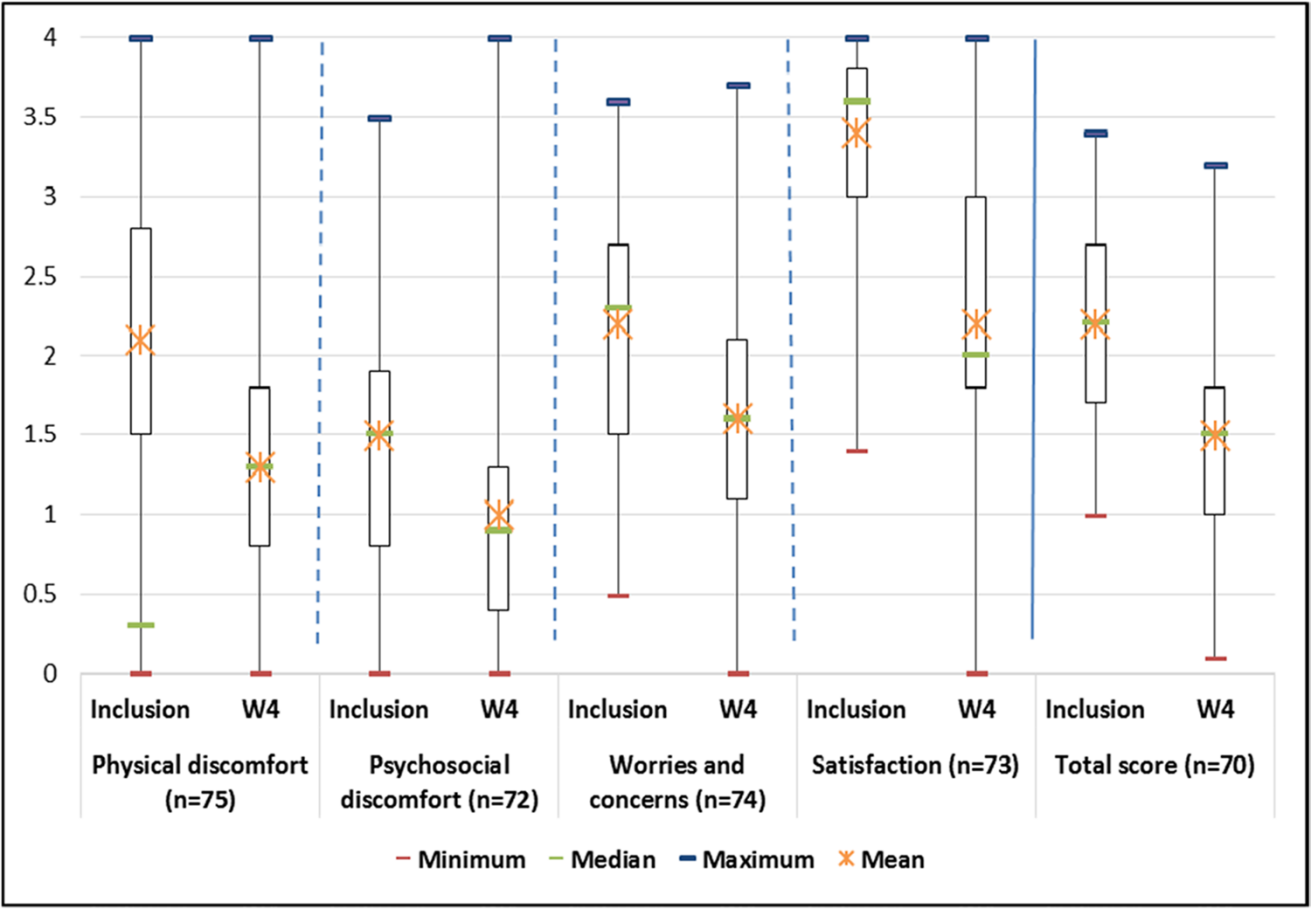# Correction to: Effectiveness of naloxegol in patients with cancer pain suffering from opioid‑induced constipation

**DOI:** 10.1007/s00520-021-06455-8

**Published:** 2021-08-13

**Authors:** Antoine Lemaire, Yoann Pointreau, Bérengère Narciso, François‑Xavier Piloquet, Viorica Braniste, Jean‑Marc Sabaté

**Affiliations:** 1 Oncology and Medical Specialties Department, Valenciennes General Hospital, Valenciennes, France; 2 Oncology and Radiotherapy Department, Inter-regional Institute of Oncology (ILC) – Jean Bernard Center, Le Mans, France; 3 Medical Oncology Department, Bretonneau Regional University Hospital Center, Tours, France; 4 Oncology and Medical Specialties Department, West Oncology Institute – René Gauducheau Center, St. Herblain, France; 5 Medical Department, Kyowa Kirin Pharma, Neuilly‑sur‑Seine, France; 6 Gastroenterology and Digestive Oncology Department, Avicenne Hospital, AP-HP, Bobigny, France; 7 INSERM U‑987, Pathophysiology and Clinical Pharmacology of Pain, Ambroise Paré Hospital, Boulogne‑Billancourt, France

**Correction to: Supportive Care in Cancer**

10.1007/s00520-021-06299-2

The article "Effectiveness of naloxegol in patients with cancer pain suffering from opioid‑induced constipation", written by Lemaire, A., Pointreau, Y., Narciso, B., Piloquet, F.-X., Braniste, V., and Sabaté, J.-M., was originally published electronically on the publisher’s internet portal on 24 May 2021 without open access. With the author(s)’ decision to opt for Open Choice the copyright of the article changed on 22 July 2021 to © The Author(s) 2021 and the article is forthwith distributed under a Creative Commons Attribution 4.0 International License, which permits use, sharing, adaptation, distribution and reproduction in any medium or format, as long as you give appropriate credit to the original author(s) and the source, provide a link to the Creative Commons licence, and indicate if changes were made. The images or other third party material in this article are included in the article’s Creative Commons licence, unless indicated otherwise in a credit line to the material. If material is not included in the article’s Creative Commons licence and your intended use is not permitted by statutory regulation or exceeds the permitted use, you will need to obtain permission directly from the copyright holder. To view a copy of this licence, visit http://creativecommons.org/licenses/by/4.0.

Figures 2 and 3 in the original are incorect, the correct figures are shown below: